# Membrane and lipid metabolism plays an important role in desiccation resistance in the yeast *Saccharomyces cerevisiae*

**DOI:** 10.1186/s12866-020-02025-w

**Published:** 2020-11-10

**Authors:** Qun Ren, Rebecca Brenner, Thomas C. Boothby, Zhaojie Zhang

**Affiliations:** 1 Department of Zoology and Physiology, University of Wyoming, 1000 E. University Ave, Laramie, WY 82071 USA; 2 Department of Molecular Biology, University of Wyoming, Laramie, WY 82071 USA

**Keywords:** Anhydrobiosis, Desiccation tolerance, Endoplasmic reticulum, Lipid droplets, Lipid metabolism, Mitochondria, Vacuole

## Abstract

**Background:**

Anhydrobiotes, such as the yeast *Saccharomyces cerevisiae*, are capable of surviving almost total loss of water. Desiccation tolerance requires an interplay of multiple events, including preserving the protein function and membrane integrity, preventing and mitigating oxidative stress, maintaining certain level of energy required for cellular activities in the desiccated state. Many of these crucial processes can be controlled and modulated at the level of organelle morphology and dynamics. However, little is understood about what organelle perturbations manifest in desiccation-sensitive cells as a consequence of drying or how this differs from organelle biology in desiccation-tolerant organisms undergoing anhydrobiosis.

**Results:**

In this study, electron and optical microscopy was used to examine the dynamic changes of yeast cells during the desiccation process. Dramatic structural changes were observed during the desiccation process, including the diminishing of vacuoles, decrease of lipid droplets, decrease in mitochondrial cristae and increase of ER membrane, which is likely caused by ER stress and unfolded protein response. The survival rate was significantly decreased in mutants that are defective in lipid droplet biosynthesis, or cells treated with cerulenin, an inhibitor of fatty acid synthesis.

**Conclusion:**

Our study suggests that the metabolism of lipid droplets and membrane may play an important role in yeast desiccation tolerance by providing cells with energy and possibly metabolic water. Additionally, the decrease in mitochondrial cristae coupled with a decrease in lipid droplets is indicative of a cellular response to reduce the production of reactive oxygen species.

## Background

Water is essential for all life and required for the majority of the biological processes. At cellular level, water plays a pivotal role in proper protein folding and the assembly of phospholipids into biological membranes. Nevertheless, certain organisms called anhydrobiotes are capable of surviving extreme water loss for a long period of time. Anhydrobiotes are widespread, having been found in the plant, animal (such as tardigrades and nematodes), fungal (such as the budding yeast *Saccharomyces cerevisiae* and other non-conventional yeasts), and prokaryotic kingdoms of life [1]. Studies have shown that many anhydrobiotes possess large quantities of non-reducing sugars (mostly disaccharides, such as trehalose) [2], intrinsically disordered proteins (IDPs) [3, 4] and heat shock proteins [5]. It is believed that these non-reducing sugars and IDPs help stabilize and preserve both membrane and protein structure and therefore mitigate the desiccation-induced stresses [6–9]. In budding yeast, dehydration causes dramatic structural changes to almost all major organelles [10–12]. Cell wall components were altered and branched mannoprotein fibrils that are similar to flocculent yeast were observed on the surface of the cell [13]. Plasma membrane invaginations occur in response to significant decrease of cell size [14]. Structural changes of the nuclear membrane and condensation of both nuclear and mitochondrial DNA has also been reported [13, 14]. Other significant changes include vacuoles, lipid droplets and peroxisomes [12]. Some of the changes are caused by oxidative stress during dehydration [15], when the oxidation level increases significantly [16]. Multiple pathways have been evolved in yeast to mitigate the oxidative damages caused by desiccation [15].

The desiccation tolerance of anhydrobiotes is typically not innate, but acquired through some ‘low’ level stress that may or not be related to desiccation. For example, in plants acquisition of desiccation tolerance is triggered by dehydration and an increase in the plant hormone abscisic acid [6, 17]. In tardigrade and *Saccharomyces cerevisiae*, desiccation tolerance is induced by starvation [4, 18]. Dividing yeast cells growing in glucose have a minimum tolerance to desiccation [18]. Cells growing in ethanol-containing medium also has a decreased desiccation tolerance, possibly due to their high degree of unsaturated fatty acids [19]. Many other factors, such as, cell growth phase, cell cycle stage, growth conditions, and conditions of dehydration-rehydration etc., also affect the desiccation tolerance. Starvation stress leads to significant physiological and morphological changes in yeast, such as a reduced metabolic rate and thickened cell wall that is more resistant to enzymatic digestion and more thermotolerant [20]. Interestingly, a similar gene expression pattern to stationary yeast was identified in proliferating yeast that were treated with certain chemicals, heat shock or irradiation [21], suggesting that yeast may possess a universal response mechanism to different environmental stresses.

In plants, the desiccation process starts with moderate dehydration (initial loss of water) and precedes to severe dehydration (loss of almost all water). In the initial stage of dehydration, the cell volume decreases due to water loss, causing cytoplasmic components to become more crowded, which could cause protein denaturation and membrane fusion [6]. The protective disaccharides and other molecules accumulate and help forming a water shell around proteins and membrane to prevent proteins misfolding as well as phase transition of membranes. In the severe water loss stage, these disaccharides function as water substitutes to replace water molecules and preserve the protein and membrane structure [2, 6]. The disaccharides could also be vitrified, especially at low water content, forming glassy matrix to avoid the membrane phase transition during dehydration [12, 22–24]. However, protein misfolding has been observed in desiccated yeast [5], suggesting that not all proteins, or membrane are protected. It is unknown whether ‘essential’ proteins and membrane structure (such as nuclear membrane) are preferentially protected.

During the desiccation process, intracellular water is still critical to maintain enzyme activities necessary to adapt to water loss. Enzyme activities were also observed in yeast during long-term desiccation (up to 180 days) when trehalose is degraded by trehalases [5]. Little is known about how trehalases remain active in this highly dehydrated state, suggesting that more mechanisms are involved in response to desiccation stress and desiccation tolerance. Not surprisingly, the necessity of several mechanisms to confer desiccation tolerance has also been reported in other systems [6].

In this study, transmission electron microscopy (TEM) was used to examine the ultrastructural changes of yeast during the desiccation process. Because stationary cells are more desiccation tolerant than the log-phase cells, we speculated that the morphological and physiological changes of stationary cells, in particular, the increased endoplasmic membrane, decrease in vacuoles and mitochondria and modulation of lipid droplet number may play an important role in desiccation tolerance of the yeast.

## Results

### Desiccation causes significant structural changes

Desiccation causes cellular damages even in anhydrobiotes [25]. In budding yeast, studies have shown that desiccation causes changes of the cellular structure, such as cell wall, plasma membrane, nucleus, vacuole, lipid droplets, peroxisomes and mitochondria (for reviews, see [10–12]). Here, transmission electron microscopy (TEM) was employed to examine and compare the ultrastructure of desiccated yeast cells in log- (desiccation-sensitive) and stationary- (desiccation tolerant) phases. First, we examined the desiccation progress of yeast cells in the 15 ml glass tubes (see Method section for details). Cells in the tubes were weighted during the desiccation process for weight loss, which was not observed 3.5 days (~ 80 h) after desiccation, indicating that cells were fully desiccated after 3.5 days of desiccation. To check the remaining amount of extracellular water, the 3.5-day-desiccated cells were placed in a 65 °C oven for 1 h, then to a 125 °C oven for 15 min [26]. A 7% weight loss was observed after oven incubation, suggesting that the desiccated cells contained about 7% extracellular water.

**Fig. 1:**
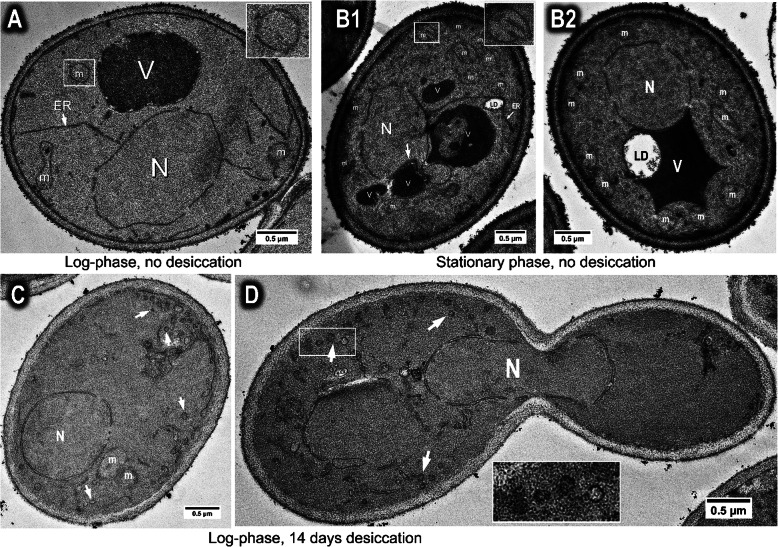
Ultrastructure of yeast cells before and after 14-day desiccation. **a** Wild type cells growing in YPD medium for overnight to log-phase, then processed for TEM observation without drying. **b** Wild type cells growing in YPD medium for 3 days to stationary phase, then processed for TEM without drying, showing abundance of mitochondria, the nucleus-vacuole junctions (arrows) and multiple vacuoles (B1), and single vacuole (B2). **c** Wild type cells growing in YPD medium for overnight to log-phase, then dried for 14 days, showing circular ER membrane (arrows), and increased gap between inner and outer nuclear membrane. **d** Wild type cells growing in YPD medium for overnight to log-phase, then dried for 14 days, showing a dividing mother (left) and daughter cell (right). ER membrane forms circular structure (arrow) but mostly observed in the mother cell. N = nucleus, V = vacuole, LD = lipid droplet; m = mitochondrion. Inset shows the enlarged image of the boxed area in the same image

Staining with potassium permanganate, which provides enhanced staining of cellular membranes [27], revealed a typical ultrastructure of overnight-grown (log-phase) yeast cells without desiccation (Fig. 1a). Stationary cells (3-day culture) without desiccation had more mitochondria than log-phase cells and the cristae of these mitochondria were more prominent (Fig. 1b). Additionally, hydrated stationary-phase cells possess one or a few dark-stained vacuoles and the vacuoles are often associated with the nucleus, forming the nucleus-vacuole junctions [28], which function as sites for piecemeal microautophagy that degrades portions of the yeast nucleus during starvation [29] (Fig. 1b). When cells were stored in the desiccated state for 14 days, significant structural changes were observed in both log- and stationary phase cells. In dried desiccation-sensitive log-phase cells, vacuoles diminished in almost all cells. ER membranes became curled, forming small circular structure (whorls), especially near the plasma membrane. These whorls were much smaller that observed in stationary desiccated cells (see next section). The gap between the inner and outer nuclear membrane expanded significantly (Fig. 1c). This phenomenon was also observed in dividing cells during log-phase growth. Circular ER membrane structures were mostly observed in the mother, but not the daughter cell (Fig. 1d). Desiccation also caused dramatic structural changes in desiccation-tolerant stationary cells, including increased ER membrane and reduced vacuole number [18]. These changes are the major focus of this study and are described in details in the following sessions.

### Desiccation triggers endoplasmic reticulum (ER) stress and unfolded protein response (UPR)

**Fig. 2:**
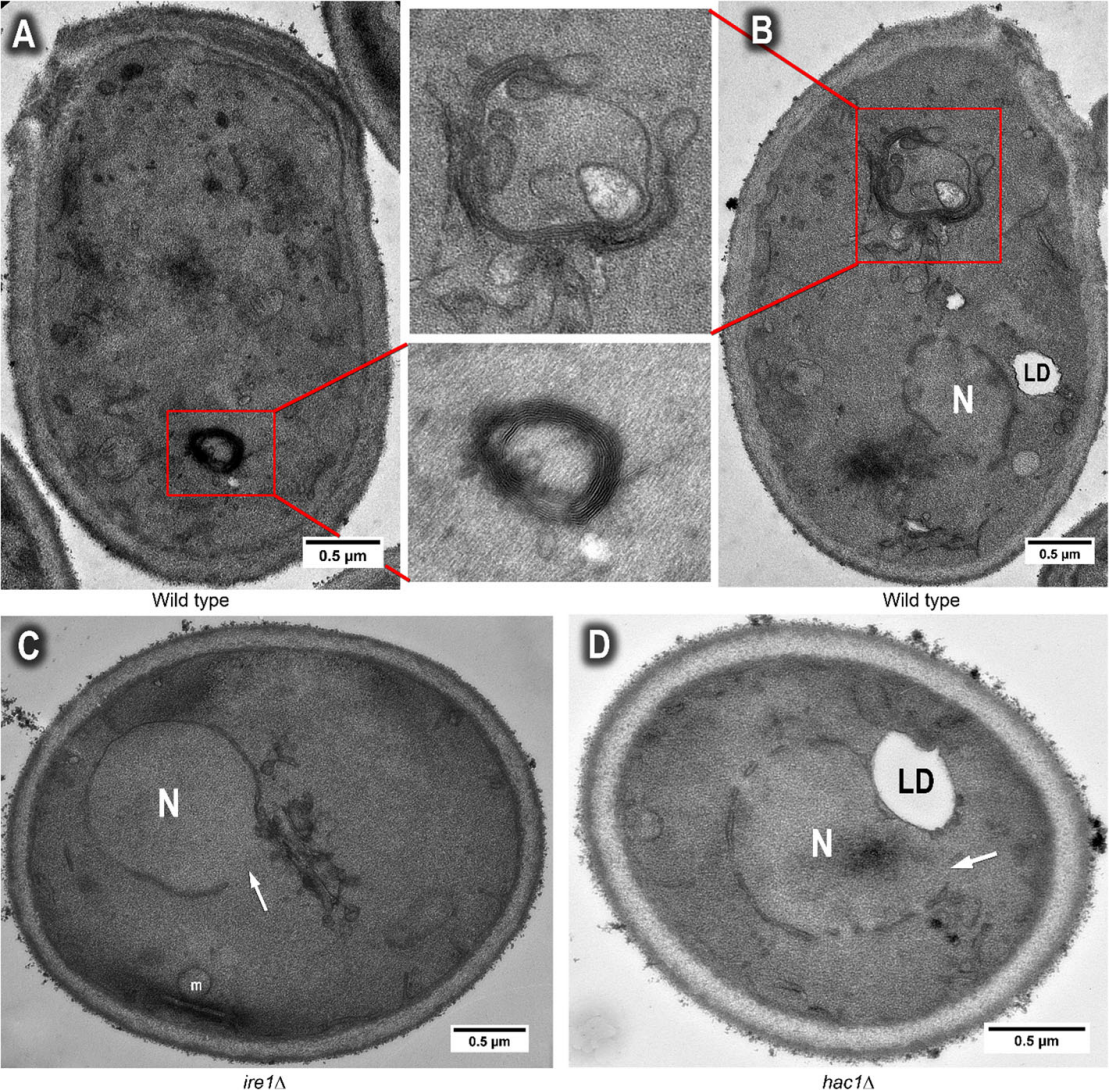
Endoplasmic reticulum (ER) whorl and whorl-like structure in desiccating cell. **a** Wild type cells growing in YPD medium for 3 days to stationary phase, then dried for 14 days, showing the endoplasmic reticulum (ER) whorl structure; **b** A whorl-like ER structure in cells at the same growing conditions as in (A). **c** *ire1Δ* mutant growing in YPD medium for 3 days to stationary phase, then dried for 14 days, showing the disorganized ER membrane around the nucleus, and the opening of the nucleus (arrow). **d** *hac1Δ* mutant growing in YPD medium for 3 days to stationary phase, then dried for 14 days, showing the opening of the nucleus (arrow). N = nucleus, LD = lipid droplet

It has been reported that misfolded and unfolded proteins trigger endoplasmic reticulum (ER) stress, which in turn, induces unfolded protein response (UPR) in yeast [30]. When treated with the ER stressor dithiothreitol, the ER forms multi-layered, ring-shaped ER whorls [30, 31]. ER whorls and whorl-like ER structure were also observed in stationary-phase yeast desiccated for 14 days (Fig. 2a, b), suggesting desiccation causes accumulation of unfolded/misfolded proteins [5], which induces ER stress, ultimately triggering UPR.

In budding yeast, UPR is mediated by the transmembrane endoribonuclease Ire1. Activation of Ire1 cleaves the mRNA of HAC1, which binds to a DNA sequence called UPR element to increase the expression of UPR target genes [32]. To further examine whether the ER whorls are related to UPR, we compared the cross sections of wild type, *ire1Δ* and *hac1Δ* mutants desiccated for 14 days. About 200 cell cross sections of each strain (from 3 independent experiments, 60–70 cells per experiment) were examined. ER whorls were observed in about 13% of wild type cells, but no ER whorls were observed in *ire1Δ* or *hac1Δ* mutant (Fig. 2c, d), further suggesting the ER whorls observed in desiccated cells (Fig. 2a, b) were induced by UPR during desiccation.

### Desiccation causes expansion of endoplasmic reticulum

**Fig. 3:**
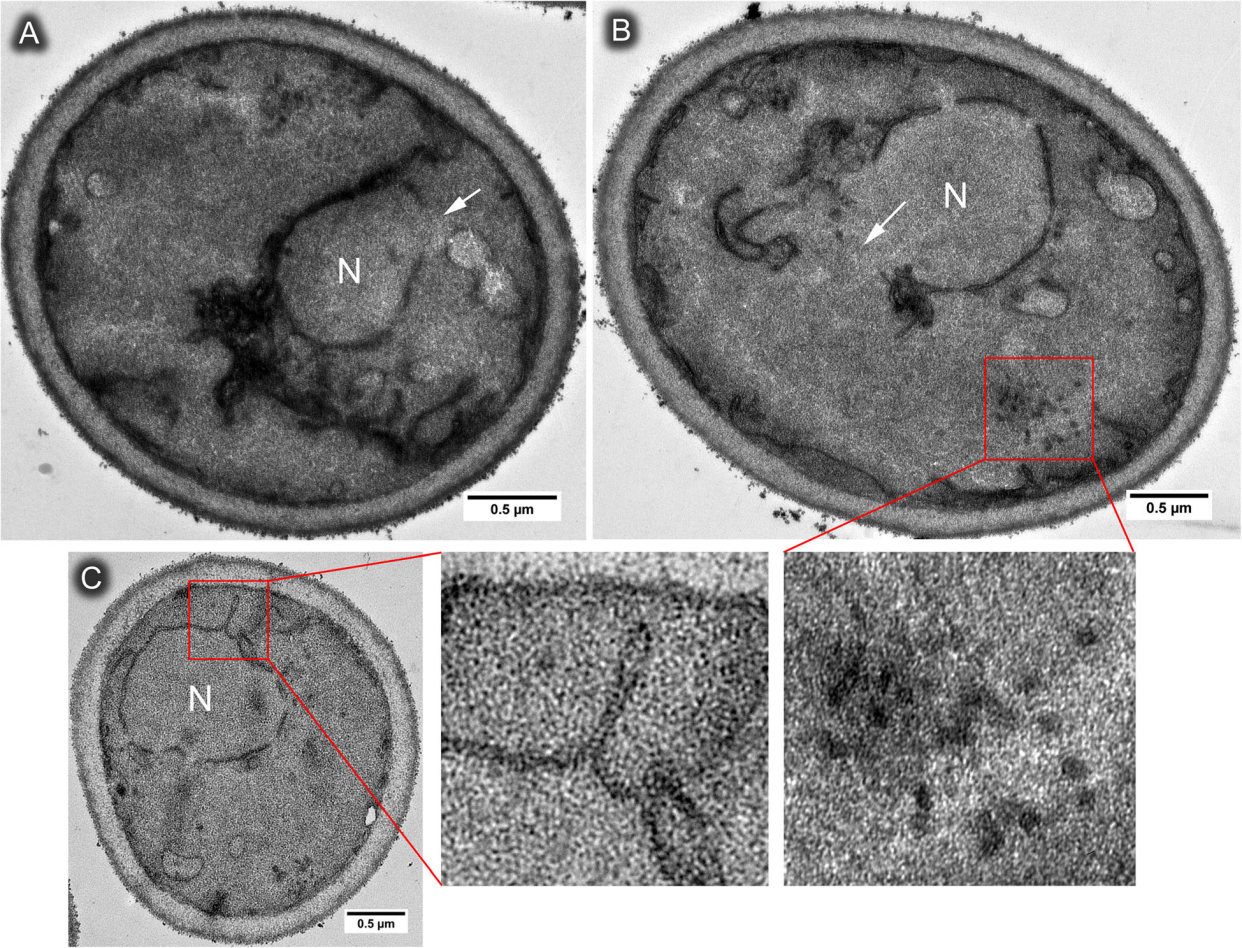
Rupture and folding of the nuclear membrane caused by desiccation. Wild type cells growing in YPD medium for 3 days to stationary phase, then dried for 14 days, showing the nuclear membrane rupture (arrow in **a** and **b**), circular ER structure (boxed area in **b**), and outward folding of the nuclear membrane (boxed area in **c**). N = nucleus

ER stress induces expansion of ER membrane and this expansion requires UPR signaling [33]. When 3-day stationary cells were desiccated for 14 days, a significant increase of ER membrane was observed in yeast cells with the ER membrane appearing to be disorganized and tangled around the nuclear membrane (Fig. 3a). This expanded and disorganized membrane was not observed in 3-day stationary cells prior to desiccation (Fig. 1b), nor in desiccated log-phase cells (Fig. 1c). These expanded membrane was different from the ER whorls, which were more organized and usually not associated with nuclear membrane. In desiccated stationary-phase cells, the ER membrane close to plasma membrane was often observed broken into small pieces, forming circular structures of 30–50 nm in diameter (Fig. 3b). One possibility is that the circular membrane structures are cross-sections of the ER whorls, or whorl-like structure observed in Fig. 2a and b. These circular structure was rarely observed in *ire1Δ* or *hac1Δ* mutant.

Much less ER membrane accumulation, including both the disorganized membrane near nucleus and the circular membrane near plasma membrane, was observed in stationary-phase *ire1Δ* or *hac1Δ* mutant (Fig. 2c, d). We measured 6 cross sections from each strain (wild type, *ire1Δ* or *hac1Δ*) that contain the disorganized membrane near nucleus. It showed an approximately 40% less membrane accumulation in *ire1Δ* or *hac1Δ* mutant than in wild type. This reduced membrane expansion may be caused by blocking of UPR signaling in *ire1Δ* or *hac1Δ* mutant or be due to the fact that both *ire1Δ* and *hac1Δ* are involved in in phospholipid biosynthesis [34].

The nuclear and plasma membrane also underwent dramatic changes during desiccation in stationary-phase cells. Outward folding of the nuclear membrane was observed (Fig. 3c). This was not observed in desiccated log-phase cells and thus may be an adaptive response mounted by stationary-phase cells to cope with dehydration-induced cell shrinkage.

Another prominent feature of the 14-day desiccated stationary cells is the rupture of the nuclear envelop. The nuclear envelop opening was always associated with disorganized ER membrane (Fig. 3a, b). Similar to the wild type, broken nuclear membrane was also observed in desiccated stationary-phase *hac1Δ* and *ire1Δ* mutants (Fig. 2c, d). Examination of about 200 cells from TEM cross sections of each strain/growth condition (from 3 independent experiments, 60–70 cells per experiment) showed that 6–8% of cells displayed nuclear opening in desiccated wild type stationary cells or *hac1Δ* and *ire1Δ* cells, but not in desiccated log-phase cells (Fig. 1c, d).

### Unfolded protein response does not affect the desiccation tolerance

**Fig. 4:**
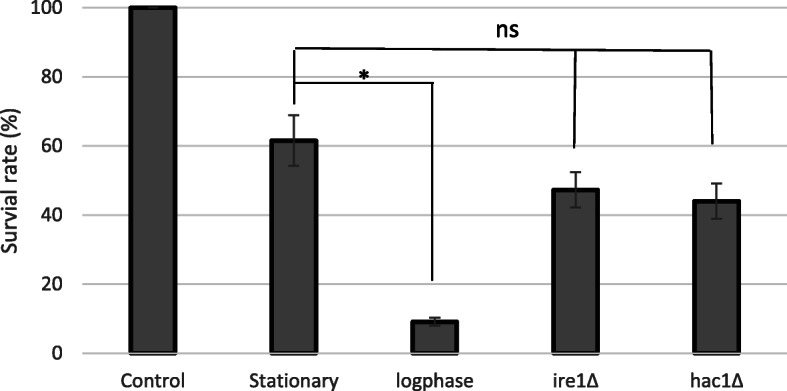
Survival rates under different growth/desiccation conditions. Control: Wild type cells growing in YPD medium for 3 days without desiccation. **Stationary:** Wild type cells growing YPD in medium for 3 days then desiccated for 14 days. **Log-phase:** Wild type cells growing in YPD medium for overnight then desiccated for 14 days. ***ire1Δ*:** *ire1Δ* mutant cells growing in YPD medium for 3 days then desiccated for 14 days. ***hac1Δ*:** *hac1Δ* mutant cells growing in YPD medium for 3 days then desiccated for 14 days. Data were presented as mean ± standard derivation (represented by the error bars) from 3 independent experiments (three plates were used in each experiment). *: *p* < 0.05; ns: not significant compared to stationary cells

We next checked the survival rates of yeast after 14 days of desiccation. The survival rate of the stationary wild type yeast was about 60%, but only 10% for log-phase cells. Interestingly, the survival rates of both *ire1Δ* and *hac1Δ* were slightly lower that the wild type, but the difference was not statistically significant (*p* > 0.05) (Fig. 4), suggesting that inactivation of UPR does not affect desiccation tolerance.

### Vacuoles diminish during desiccation

The yeast vacuole is the largest organelle in a yeast cell and has similar functions to the lysosome in higher eukaryotes or the plant vacuole. In yeast the vacuole serves as the primary site for protein degradation and recycling, especially during starvation. Using TEM, we observed that hydrated 3-day stationary cells usually have one or a few dark-stained vacuoles (Fig. 1b). Examination of about 200 cells from TEM cross sections (from 3 independent experiments) showed that 14 days after desiccation, over 90% of the stationary cells had no vacuoles (Figs. 2a, b and 3). When observed, the vacuoles were lightly stained (Fig. 6a). Vacuoles were also absent in most of the log-phase desiccated cells (Fig. 1c, d).

### Structural changes during the desiccation process

**Fig. 5:**
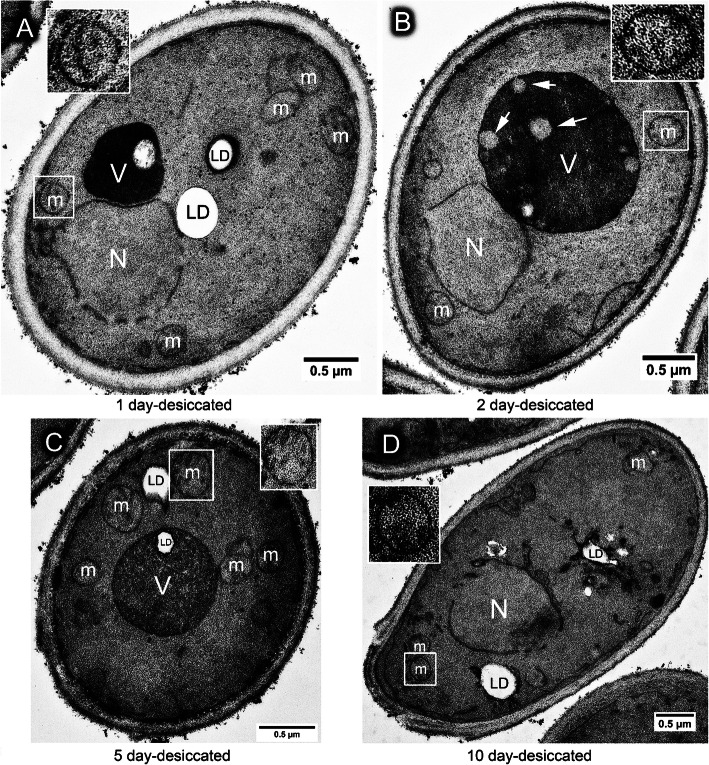
Ultrastructural changes of yeast cells after desiccated for 1 to 10 days. **a** Wild type cells growing in YPD medium for 3 days, then dried for 1 day, showing a similar structure to non-desiccated cells as in Fig. 1b. **b** Wild type cells growing in YPD medium for 3 days, then dried for 2 days, showing less cristae in mitochondria and light-stained spots in vacuole (arrows). **c** Wild type cells growing in YPD medium for 3 days, then dried for 5 days, showing the light-stained spot in vacuole (outline arrow), and the engulfment lipid droplets by the vacuole. **d** Wild type cells growing in YPD medium for 3 days, then dried for 10 days, showing ER membrane surrounded lipid droplets. N = nucleus, V = vacuole, LD = lipid droplet, m = mitochondrion

Next, we evaluated the ultrastructural changes during desiccation by examining stationary-phase yeast cells dried for 1, 2, 5 or 10 days. No significant difference was observed between cells dried for 1 or 2 days (Fig. 5a), in which nucleus was usually intact, similar to pre-desiccated cells, except that the mitochondrial cristae became less obvious. Vacuoles were mostly dark-stained, but light-stained spots were often observed inside the vacuoles (Fig. 5b), suggesting a possible initiation point for vacuole degradation. More light-stained vacuoles were observed in 5-day desiccated cells. Observation of about 200 cell cross sections from 3 independent experiments showed that engulfment of lipid droplet occurred in about 9–10% of either 2-day or 5-day-dried cells (Fig. 5c) and vacuoles were absent in more than 50% of 10-day desiccated cells and some lipid droplets were surrounded by ER membrane (Fig. 5d).

### Endoplasmic reticulum (ER) and lipid droplets (LDs) dynamic in desiccated yeast cells

**Fig. 6:**
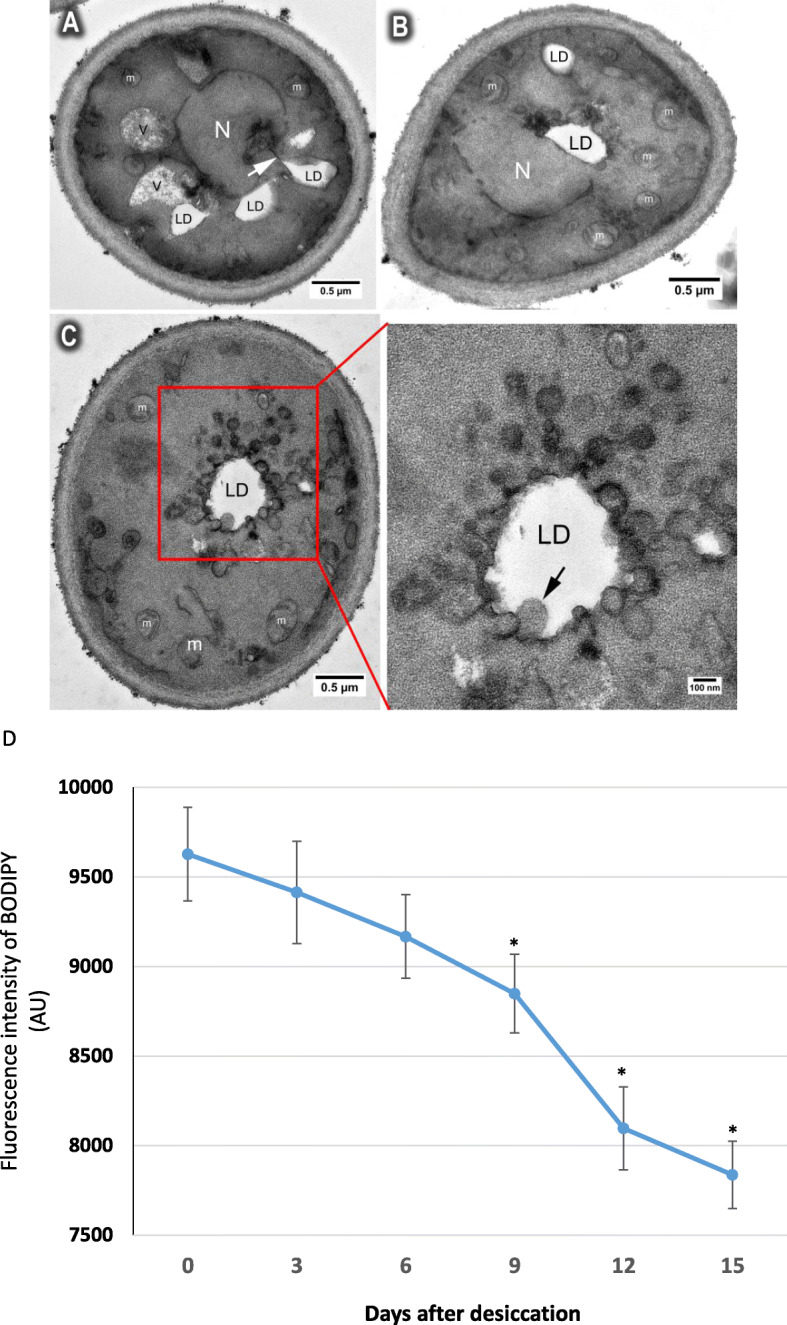
Endoplasmic reticulum (ER) and lipid droplets (LDs) dynamic in desiccated yeast cells. **a** Wild type cells growing in YPD medium for 3 days, then dried for 14 days, showing LD associated with ER membrane (arrow). **b** Wild type cells growing in YPD medium for 3 days, then dried for 14 days, showing LD near the nucleus is surrounded by disorganized ER membrane. **c** Wild type cells growing in YPD medium for 3 days, then dried for 14 days, showing the LD surrounded by circular ER structures. Some of the circular structures appear to have no membrane (arrow in boxed area). **d** Wild type cells growing in YPD medium for 3 days, then dried for 0 to 15 days. The survival rates were measured every 3 days using plate assay. Data were presented as mean ± SD from 3 independent experiments. *: *p* < 0.05, compared to fluorescence intensity of 0 day. N = nucleus, V = vacuole, LD = lipid droplet, m = mitochondria

**Fig. 7:**
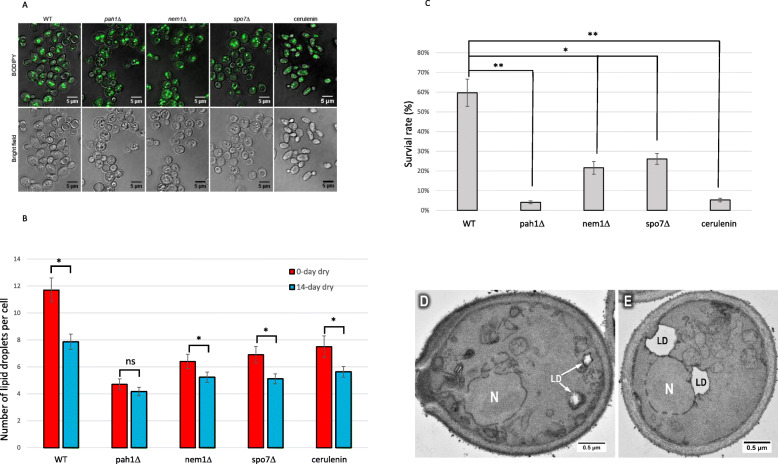
Desiccation tolerance correlate with lipid droplets. **a** Confocal microscopy images of BODIPY stained cells growing in YPD medium for 3 days prior to desiccation. **b** Quantification of LD numbers from the confocal microscopy images of prior to desiccation (red) and 14 days after desiccation (blue). **c** Cell survival rate after 14 days of desiccation. *: *p* < 0.05, **: *p* < 0.01, ns: not statistically significant. Cerulenin: 4 μg/ml was added into the culture medium prior to the 3-day culture. Data in (**b**) and (**c**) were presented as mean ± SD (represented by error bars) from 3 independent experiments. **d** *pah1Δ* cells growing in YPD medium for 3 days, then dried for 14 days, showing the lipid droplet structure. **e** *nem1Δ* cells growing in YPD medium for 3 days, then dried for 14 days, showing the lipid droplet structure. N = nucleus, LD = lipid droplet

Lipid droplets (LDs) are ER–derived neutral lipid storage organelles [35]. They are universally conserved in both prokaryotes and eukaryotes and their biogenesis primarily occurs from ER, where newly synthesized neutral lipids emerge from and are surrounded by a phospholipid monolayer [36]. During desiccation, LDs were often observed to be associated with normal (Fig. 6a), disorganized (Fig. 6b), or circular ER membrane (Fig. 6c). It was observed that some of the circular structures had no membranes (arrow in Fig. 6c), suggesting that the ER membrane might be degraded and the free fatty acids were stored in LDs. If this is the case, we speculate that the overall LDs would increase during desiccation. To test this possibility, we checked the LD levels every 3 days for 15 days during desiccation. To our surprise, the LD level significantly decreased, rather than increased during the desiccation process (Figs. 6d, 7b). We reasoned that LDs might be consumed during desiccation to provide energy. This notion is supported by the TEM observation that LDs were engulfed during early stage of the desiccation by vacuoles (Fig. 5c), which degrade LDs by a process that resembles microautophagy [37, 38].

### Defects of lipid droplet synthesis reduce desiccation tolerance

To further test the possible role of LDs in desiccation tolerance, we examined yeast strains that are defective in LD formation. The yeast Pah1 is a homologue of mammalian lipin 1 protein, which plays important roles in glycerolipid biosynthesis. It regulates lipid droplet formation and nuclear/ER membrane growth [37]. Deletion of PAH1 causes significant decrease of number of LDs [39]. BODIPY staining revealed that the number of LDs in the 3-day stationary cells of *pah1Δ* was significantly less than that in wild type either prior to or after 14 days of desiccation (Fig. 7a, b). No significant change of LDs in *pah1Δ* mutant was observed after 14 days of desiccation. The survival rate of the *pah1Δ* mutant was significantly lower than the wild type (Fig. 7c). TEM observation revealed that after 14 days of desiccation, the LDs in *pah1Δ* cells lack clear boundaries and are usually smaller than those in wild type cells. (Fig. 7d). Measurement of 50 LD diameters from TEM cross sections showed that the average LD size/diameter of *pah1Δ* was about 1/3 of the wild type.

Pah1 is regulated by the Nem1-Spo7 phosphatase complex. Deletion of NEM1 or SPO7 partially inhibits the Pah1 activity and causes a phonotype similar to *pah1Δ* [40]. When the *nem1Δ* and *spo7Δ* deletion mutants in stationary phase were dried for 2 weeks, we found that their survival rates were much lower than in wild type cells, but higher than that of the *pah1Δ* (Fig. 7c). BODIPY staining showed that the number of LDs in *nem1Δ* or *spo1Δ* was also in between the wild type and *pah1Δ* (Fig. 7a). TEM observation of the 14-day dried *nem1Δ* or *spo1Δ* cells showed a structure similar to *pah1Δ*, except that LDs were larger than that in *pah1Δ* (Fig. 7d-e). In all three mutants, much less ER membrane accumulation was observed.

Next, we used the antifungal agent cerulenin, which inhibits the biosynthesis of fatty acids and steroids, to reduce LD formation [41]. In the presence of 4 μg/ml cerulenin, the number of LDs was significantly reduced (Fig. 7a, b). After 14 days drying, the survival rate of the cerulenin treated cells was significantly lower than the non-treated control (*p* < 0.01), similar to that of the *pah1Δ* mutant (Fig. 7b), further suggesting that accumulation of LDs is important to desiccation tolerance.

## Discussion

Using luciferase and prion propagation assays, Tapia and Koshland [5] demonstrated that desiccation induces protein misfolding, which is mitigated by the presence of trehalose. Misfolded proteins cause ER stress and trigger the UPR, which in turn, induces expansion of ER membrane [33]. In this study, we observed directly with TEM that desiccation causes the formation of ER whorls, a multi-layered, ring-shaped structure that has been reported under ER stress [30, 31]. Our results show that the desiccation tolerance in *ire1Δ* or *hac1Δ* (deactivation of UPR) did not increase, but slightly decreased, suggesting that UPR may have an positive effect on desiccation tolerance by increasing the ER membrane, which may be used as a source for fatty acids as further discussed below.

One interesting phenomenon we observed in this study is the rupture of the nuclear envelop, which was often associated with the disorganized ER membrane (Fig. 3a). This phenomenon was also observed in *ire1Δ* or *hac1Δ* mutant, but not in desiccated log-phase cells, which had a much lower survival rate. It suggests that the opening of the nuclear envelop during desiccation might be a protection mechanism to mitigate the dehydration pressure that causes shrinkage of the nucleus. It cannot be ruled out that the cells with ruptured nuclear envelop are dead cells, but the fact that nuclear rupture was not observed in dried desiccation-sensitive log-phase cells suggests a putative connection to desiccation tolerance. One possibility for how this opening is generated is by the nucleus-vacuole junctions (Fig. 3b), which function as sites for piecemeal microautophagy of the nucleus [28]. During mitosis, the budding yeast nuclear membrane remains intact (termed closed mitosis), while in animals and plants, the nuclear membrane disassembles before chromosome segregation, which is termed open mitosis. The fission yeast *Schizosaccharomyces japonicas*, however, undergoes a semi-open mitosis, where the nuclear membrane partially open before chromosome segregation [42]. The opening of the nuclear membrane during desiccation resembles the semi-open mitosis in *S. japonicas* and a similar mechanism might be used to “repair” the ruptured nuclear envelope when condition allows. Another possibility for the observed nuclear membrane opening is that desiccation may cause compositional changes of nuclear membrane [19], making it difficult to be visualized by TEM using routine chemical fixation and staining [12].

The yeast vacuole plays an important role in nutrient storage, macromolecule degradation, detoxification, pH- and ion-homeostasis [43, 44]. It is a dynamic organelle that responds to cell cycle and environment, especially the availability of nutrients. Log-phase cells usually have multiple, small vacuoles, while stationary cells, or cells deprived of carbon source, have fewer but relatively large vacuoles [45]. Interestingly, we observed that the majority of the vacuoles diminished after 14 days of desiccation in stationary-phase cells (Figs. 2a, b and 3a). The degradation of vacuoles appears to be initiated as early as 2 days after desiccation (Fig. 5b). The removal of vacuoles could be an adaptive response to the decreased cell volume due to the dehydration. During the desiccation process, there is no apparent external nutrients and water available. It is possible that the vacuoles are degraded to provide energy and water for preparative activities essential for survival during early stage of desiccation.

Lipid droplet (LD), an organelle surrounded by a monolayer of phospholipid, are found in all organisms. It is the primary cellular organelle for regulating the storage and hydrolysis of neutral lipids, including triglycerides and sterol esters. In yeast, the number of LDs increases in response to nutrient depletion. As a result, stationary cells have more LDs than log-phase dividing cells. A recent report showed that the number of LDs increased even when yeast cells were given a short period of glucose starvation by replacing glucose with a non-hydrolysable glucose analogue [46]. At stationary phase, fatty acids are released slowly from triglycerides and degraded via β-oxidation, to provide energy necessary for cellular maintenance [41]. In our current study, we observed that the level of LDs significantly decreased during desiccation (Fig. 6d), suggesting that the neutral fat was consumed by the desiccating cells. Catabolism of neutral fat, such as triglycerides, can provide the necessary energy for cells, while no other nutrients available. It is also possible these lipids could be used to repair or restore the membrane damages during rehydration [12].

Metabolic water, which results from the oxidation of cellular carbohydrates, proteins or lipids, is an important alternative water source for many organisms, especially animals in desert, insects and migrating birds [47, 48]. Compared with carbohydrates and proteins, oxidation of fat produces the most amount of water (about 110 g of metabolic water per 100 g of fat) [49]. Unlike the desert animals that need the metabolic water to compensate the water insufficiency, the metabolic water, possibly from β-oxidation of the lipids, might be crucial to the survival of the desiccating yeast and keeping certain enzymes, such as trehalases, functional [5].

Another surprising finding of our study is that many lipid droplets in the desiccated cells were surrounded by circular ER membrane structures (Fig. 5c), and some of the circular membranes appeared to have no membrane. One possibility is that the phospholipid of the membrane might be converted into free fatty acids, which may be used as a source of energy and metabolic water. Another possibility is that the ER-LD contact is required to quickly break down lipids in the LDs, or to supply phospholipids for ER membrane expansion.

One limitation of our current study is the mixed population of desiccated cells, some were viable and some were dead. It is difficult to know whether the cellular changes observed by TEM are from viable or dead cells. It is ideal to improve the desiccation tolerance with near 100% viable desiccated cells. This will provide better assurance of the characteristics of viable cells.

## Conclusion

Our study demonstrated that yeast cells undergo significant structural changes in response to desiccation stress. The most prominent changes include the diminishing vacuoles, increased ER membrane, reduction in mitochondrial cristae, and dynamic changes in lipid droplet number and size. Our results suggest that the metabolism of the lipid droplets and membrane may play an important role in desiccation tolerance of the budding yeast.

## Methods

### Yeast strains and growth conditions

Yeast (*Saccharomyces cerevisiae*) cells were grown in YPD medium (1% yeast extract, 2% peptone and 2% dextrose) at 30 °C with constant shaking at 250 rpm. The wild type parent strain BY4742 (*Matα his3Δ leu2Δ met15Δ ura3Δ*) and all deletion mutants were purchased from Thermo Scientific (Waltham, MA). For deletion mutants, each gene disruption was replaced with a KanMX module [50]. For cerulenin treatment, cerulenin (Thermo Scientific, Waltham, MA) stock solution (10 mg/ml in 100% ethanol) was diluted to a final concentration of 4 μg/ml into YPD medium. For controls, the same amount of 100% ethanol was added in place of cerulenin.

### Sample preparation for transmission electron microscopy (TEM)

TEM samples were prepared as described previously [51] with the following modification. Yeast cells were cultured in YPD to log-phase (14–16 h, overnight culture) or to stationary phase (72 h, 3-day culture). All cultures were conducted at 30 °C with constant shaking (250 rpm) using 15 ml round-bottom glass tubes (Fisher Scientific, Waltham, MA) filled with 5 ml of culture medium. Cells were processed for TEM directly, or desiccated (see below) before being processed for TEM. For desiccated cells, cells were resuspended in phosphate-buffered saline (PBS) (pH 7.2), vortexed for 30 s and immediately centrifuged (× 1000 g for 5 min). Cells were then fixed in 2.5% (v/v) glutaraldehyde in PBS for 90 min at room temperature. Cells were washed twice with water and further fixed by 2% freshly prepared potassium permanganate in water for 90 min at room temperature. Fixed cells were stained with 2% uranyl acetate for 90 min, then dehydrated with 30, 50, 75, 85, 95% and 3 changes of 100% ethanol (60 min/step). Cells were transitioned with propylene oxide, infiltrated in Spur resin (Electron Microscopy Sciences, PA). The infiltration steps were 25% resin (in propylene oxide) for 4 h, 50% resin overnight, 75% resin for 4 h, then 100% resin (2 changes) overnight. The cells were treated twice (2 min/each) with a microwave (Pelco Bio-Wave 34,700, Ted Pella, CA) while in 100% resin. The settings were 650 W with a restriction temperature of 43 °C [52]. The prolonged fixation, dehydration infiltration are essential for the desiccated yeast cells. The resin was polymerized at 65 °C overnight in the oven. Ultrathin sections of 60 nm were cut with a diamond knife, stained with lead citrate, and examined using a Hitachi H-7000 electron microscope (Hitachi High-Tech America, Schaumburg, IL) equipped with a cooled, high-resolution digital camera (Gatan Inc.). Majority of images were taken at × 8000 magnification, which corresponds to a pixel size of 1.4 nm × 1.4 nm.

### Desiccation tolerance assay

Cells were grown in 15 ml round-bottom glass tubes (Fisher Scientific, Waltham, MA) to log- or stationary phase in YPD medium. Cells were centrifuged (× 1000 g for 5 min) and the culture medium was discarded. The culture tubes without caps were placed in a humid chamber (23 °C, 50% relative humility) and cells were allowed to desiccate for desired days. After desiccation, cells were resuspended in water, vortexed for 30 s, then diluted to 3000 cells/ml. 100 μl of cells were plated on YPD agar plates (1% yeast extract, 2% peptone, 2% dextrose and 2% agar) for 2–3 days, and the number of colonies were counted. Non-desiccated cells at the same growth conditions were used as controls. 3–4 plates were used for each strain or growth condition. Desiccation tolerance (survival rate)(%) was calculated as (number of colonies of desiccated cells)/(number of colonies of non-desiccated cells) X 100 from 3 independent experiments.

### Estimation of membrane expansion

ImageJ software (https://imagej.nih.gov/ij/) was used to estimate membrane expansion from TEM cross sections. Expanded membrane near the nucleus of the 14-day desiccated stationary cells was manually selected and the area was measured. Six sections of each strain (wild type, *ire1Δ* and *hac1Δ*) from 2 independent experiments was used for the estimate.

### Confocal microscopy imaging and quantification of lipid droplets

BODIPY 493 (Thermo Fisher Sci., Waltham, MA) was used to stain lipid droplets. For confocal microscopy imaging, BODIPY 493 was diluted with PBS to a final concentration of 1.25 μg/mL from its stock (1 mg/mL in 100% ethanol). Desiccated cells were briefly rehydrated and stained directly with the diluted BODIPY 493 for 5 min in the dark. Stained cells were imaged using a Zeiss 700 laser scanning confocal microscope (Zeiss, NY) with a 63X, 1.4 NA oil objective. Z-stacks of 1 μm step size were acquired and images were presented as maximum projections for fluorescence and the middle section for bright-field. For counting the number of LDs, at least 70 cells were counted from the maximum projected images of each experiment. Data were presented as mean ± standard derivation (SD) from 3 independent experiments.

For quantitative comparison of the LD fluorescence intensity, cells were first diluted and adjusted to OD_600_ = 0.1. Cells were then stained with BODIPY 493 for 5 min in the dark. The relative fluorescence intensity was measured using a Tecan infinite M200 fluorescence microplate reader (Tecan US, Morrisville, NC) with excitation/emission at 495/530 nm. Data were presented as mean ± standard derivation (SD) from 3 independent experiments.

### Statistical analysis

Data were presented as mean ± SD from 3 independent experiments. Student’s t-test was used to compare the difference between groups. *P* < 0.05 was considered as statistically significant.

## Data Availability

The datasets and original microscopic images used in the current study are available from the corresponding author on reasonable request.

## Abbreviations

IDPs: Intrinsically disordered proteins
TEM: Transmission electron microscopy
ER: Endoplasmic reticulum
UPR: Unfolded protein response
LDs: Lipid droplets
YPD: Yeast extract, peptone and dextrose
PBS: Phosphate-buffered saline
SD: Standard derivation
